# Inter-amphibian predation in the Early Cretaceous of China

**DOI:** 10.1038/s41598-019-44247-7

**Published:** 2019-05-23

**Authors:** Lida Xing, Kecheng Niu, Susan E. Evans

**Affiliations:** 1 State Key Laboratory of Biogeology and Environmental Geology, China University of Geosciences, Beijing, 100083 China; 2 School of the Earth Sciences and Resources, China University of Geosciences, Beijing, 100083 China; 3 Yingliang Stone Nature History Museum, Nan’an, 362300 China; 4 Department of Cell and Developmental Biology, University College London, Gower Street, London, WC1E 6BT England

**Keywords:** Palaeontology, Taxonomy

## Abstract

For most fossil taxa, dietary inference relies primarily on indirect evidence from jaw morphology and the dentition. In rare cases, however, preserved gut contents provide direct evidence of feeding strategy and species interaction. This is important in the reconstruction of food webs and energy flow through ancient ecosystems. The Early Cretaceous Chinese Jehol Biota has yielded several such examples, with lizards, birds, small dinosaurs, and mammals as both predator and prey. Here we describe an Early Cretaceous fossil frog specimen, genus *Genibatrachus*, that contains an adult salamander within its body cavity. The salamander is attributed to the hynobiid-like genus *Nuominerpeton*. The salamander skeleton is complete and articulated, suggesting it was caught and swallowed shortly before the frog itself died and was buried.

## Introduction

Assessing the diet of fossil organisms is important in understanding how they related to their environment and to other organisms within that environment, for example in reconstructing food-webs and energy flow within an ecosystem^1,2^. However, dietary inference in extinct organisms must usually be based on dentition and jaw architecture, by comparison with living relatives with known feeding strategies. This can be challenging, especially for reptiles and amphibians with relatively simple dentitions. Gut contents provide the best direct evidence of diet in fossil taxa, and can yield surprises. Thus, for example, the simple conical teeth of the Early Cretaceous Chinese lizard *Yabeinosaurus* suggested insectivory^1^, but gut contents show it to have been a frequent piscivore^3^, indicating a rather different set of ecological interactions. Nonetheless, preserved and identifiable gut contents are relatively rare, especially in small tetrapods.

Invertebrate remains (insects, conchostracans) have been recorded in the guts of fossil salamanders from the Jurassic of China^4,5^, and indeterminate gut contents were noted in a salamander from the Early Cretaceous Spanish locality of Las Hoyas^6^. In fossil frogs, the record of gut contents is also most confined to fragmentary remains of insects, sponge or snail shell fragments, and plants^7–9^. Fossil evidence of amphibian predation on vertebrates is much rarer. The only example we are aware of in a salamander is that of a 40–35 myr (Eocene) specimen of *Phosphotriton sigei* from France that contains frog remains^10^. In frogs, recorded vertebrate remains include fish bones in an Oligocene palaeobatrachid from Germany^11^, indeterminate reptile bones in an Eocene frog from Messel, Germany^12^, and vertebrae of a larval frog in a specimen of the semi-aquatic Miocene *Rana pueyoi* from Spain^8^. Here we add to the record for frogs, with a specimen from the Early Cretaceous of China that has a complete adult salamander in its gut.

## Results

### Locality and horizon

**Figure 1:**
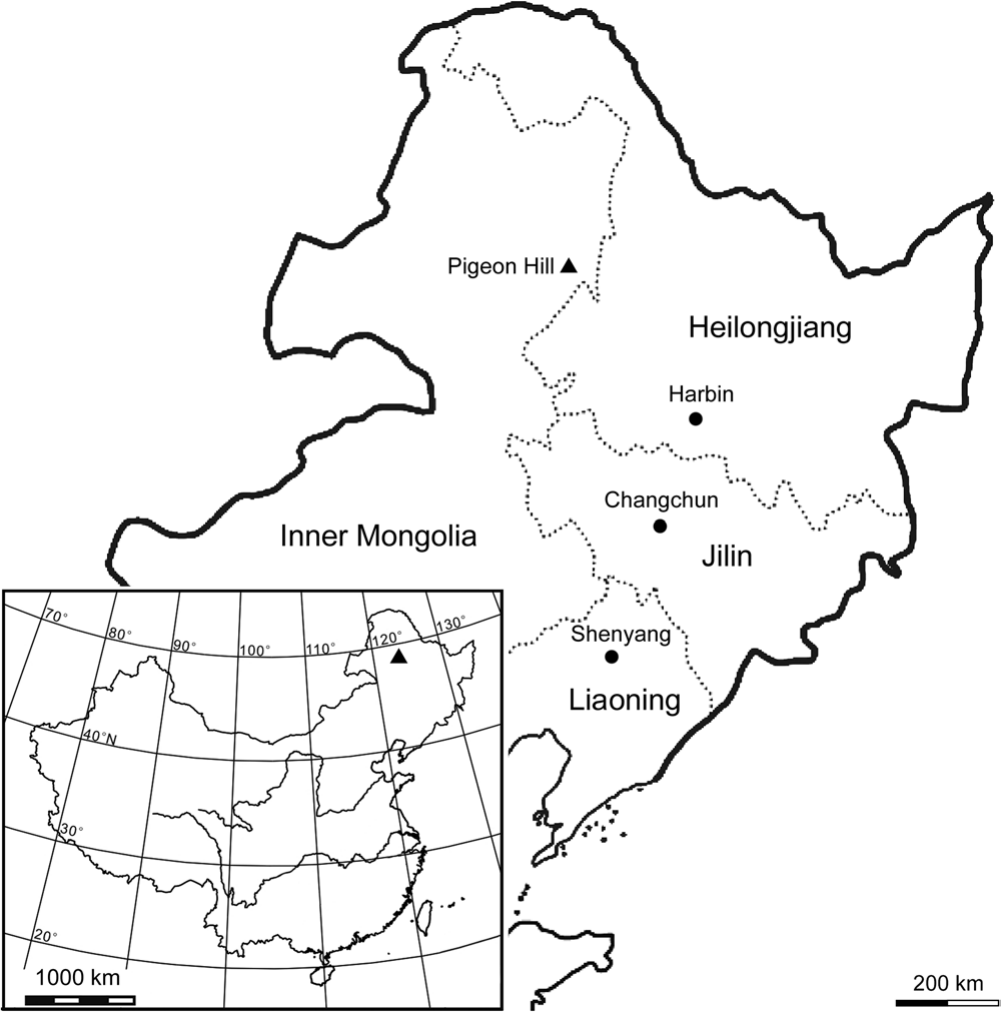
Map of China showing the position of the Early Cretaceous Pigeon Hill locality in Inner Mongolia.

The specimen, Yingliang Stone Nature History Museum (YLSNHM), YLSNHM01088, is represented by a part and counterpart block recovered from the Pigeon Hill locality, near Taipingqiao Village, Baoshan Town, Morin Dawa Daur Autonomous Banner of Hulunbuir City, Inner Mongolia, China (Fig. 1). The deposits from which it was found are those of the Guanghua Formation that has been dated at 120–125 Ma^13^, and are thus stratigraphically and chronologically equivalent to the main fossil bearing beds of Yixian Formation in western Liaoning Province (Ar40/Ar39 dating^14^).

### Description and interpretation

**Figure 2:**
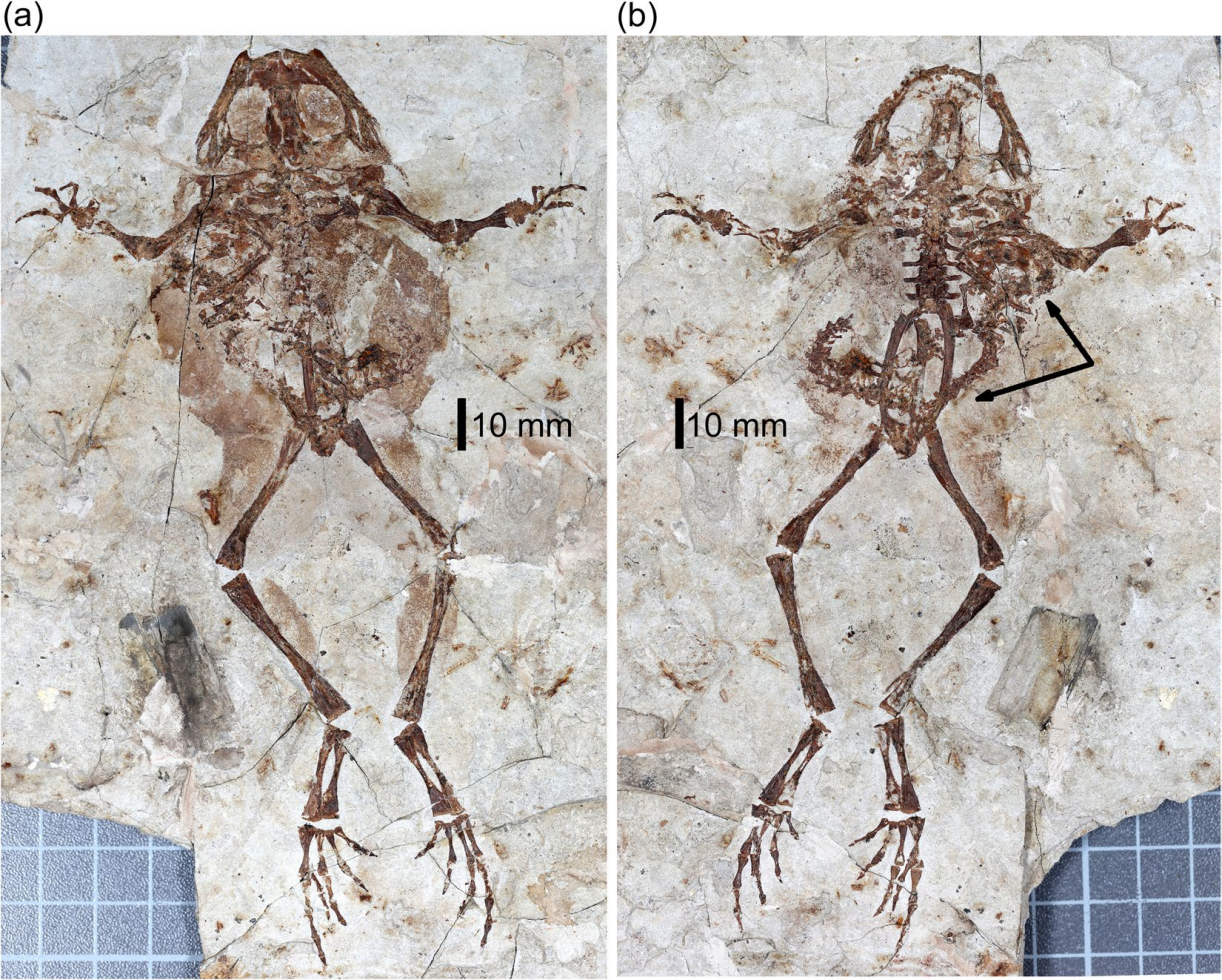
Specimen YLSNHM01088, a frog of the species *Genibatrachus baoshanensis* containing the skeleton of a salamander, cf. *Nuominerpeton*, in the body cavity. (**a**) Main part, skeleton in dorsal view, and (**b**) counterpart block. The majority of the salamander skeleton is on the counterpart block (**b**).

Specimen YLSNHM01088 preserves the skeleton of a medium-sized frog (Snout-Pelvis length [SPL] = 73.6 mm, skull length 24 mm) in dorsal view (main block) with the soft tissue outline of the body clearly visible (Fig. 2a,b). The frog skeleton matches the description given of *Genibatrachus baoshanensis*^15^, a pipanuran frog previously recorded from the Pigeon Hill locality, in the presence of unsculptured skull bones, eight presacral vertebrae, procoelous vertebral centra, free ribs on presacral vertebrae 2–4, short, slender, fused ribs on presacrals 5–8, unexpanded sacral diapophyses, sickle-shaped clavicle with a lateral spike, coracoid with expanded proximal and distal ends, relatively short forelimbs (40% of hind limb length), a tibiofibula that is longer (115%) than the femur, and slender, unfused tibiale and fibulare of which the latter is slightly longer. The skull is broad, and each maxilla carries around 50 small closely spaced teeth (40 in the holotype^15^). The holotype specimen is also described^15^ as showing a stout robust body outline. This is consistent with specimen YLSNHM01088 where the well-preserved soft tissue outlines portray a heavily built frog with a broad body and thick, presumably strongly muscled, thighs and crura.

The holotype of *G. baoshanensis* was recorded as having an SPL of 70 mm and a skull length of 23 mm^15^. Specimen YLSNHM01088 is slightly larger. However, it appears to be less skeletally mature than the holotype, in that the ends of the long bones are unfinished and lack ossified articular surfaces. In extant frogs, individuals of one gender (usually female) are often larger than the other so it is possible that whereas the holotype was skeletally mature, specimen YLSNHM01088 was of a different gender and still growing.

**Figure 3:**
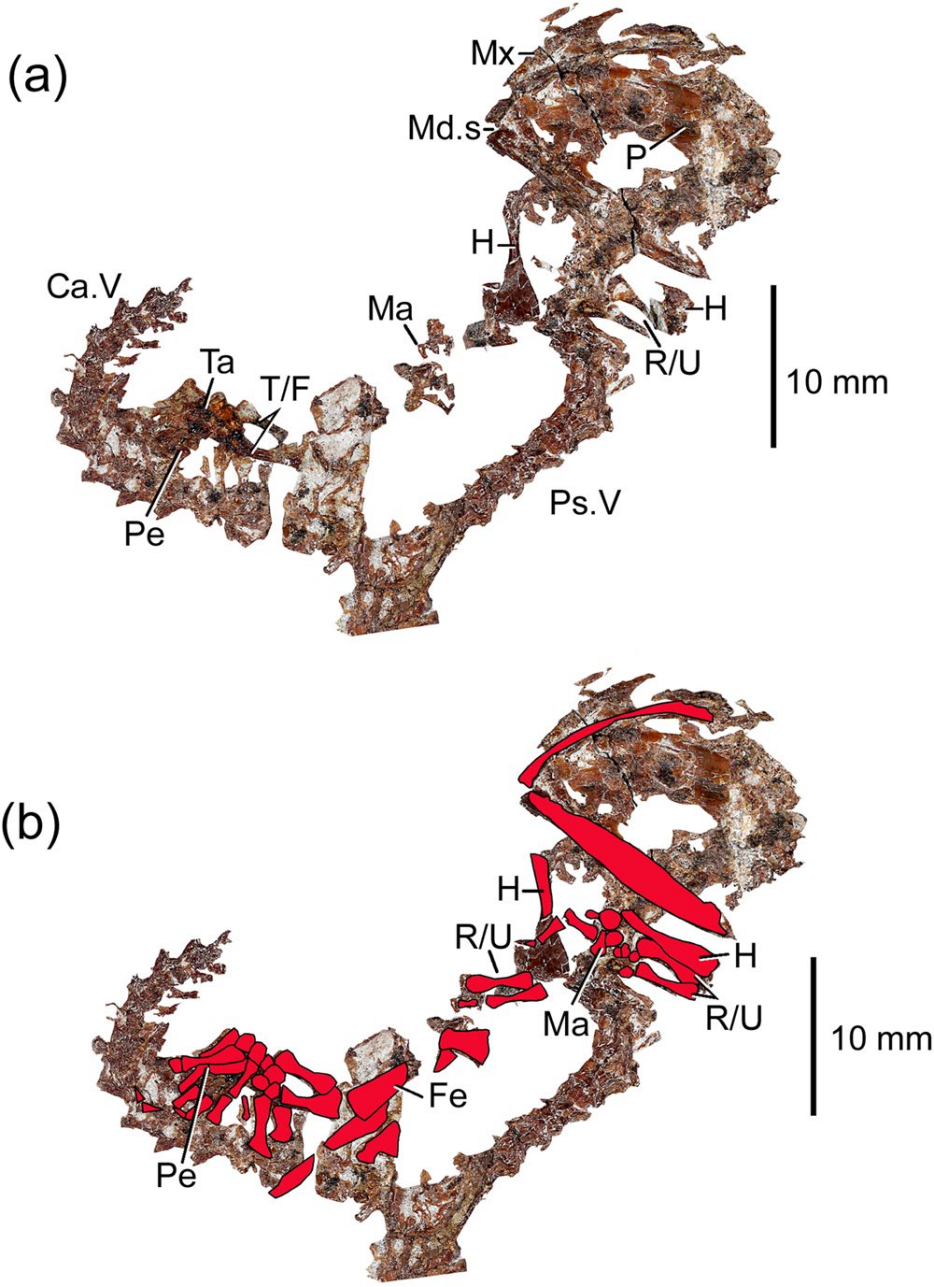
Specimen YLSNHM01088. (**a**) The skeleton of the salamander cf. *Nuominerpeton*, in ventral aspect, extracted from the counterpart block. (**b**) As (**a**), but with limb and jaw elements from the main block superimposed in red. Abbreviations: Ca.V, caudal vertebrae; Fe, femur; H, humerus; Ma, manus; Md.s, mandibular symphysis; Mx, maxilla; P, parietal; Pe, pelvis; Ps.V, presacral vertebrae; R/U, radius and ulna; Ta, tarsus; T/F, tibia and fibula.

The salamander skeleton in the frog’s gut is most clearly visible on the counterpart block, where the skull, vertebral column, and some parts of the fore- and hindlimbs are preserved (Fig. 3a). More of the limb elements are preserved on the main block (Fig. 3b). The salamander skeleton extends from under the frog’s anatomical left shoulder girdle (skull), along the frog’s left flank (forelimbs and anterior spine), and across the frog’s pelvic region ventral to the ilia and urostyle. The salamander’s tail curls up along the right side of the frog’s abdomen but the distal end is missing. The head is twisted in relation to the vertebral column so that the salamander skull, in ventral view, lies at roughly 90 degrees to the vertebral axis with the jaw symphysis close to the frog’s 5^th^ and 6^th^ presacral vertebrae (Fig. 2b).

The only salamander currently known from the Pigeon Hill locality is *Nuominerpeton aquilonaris*^16^, a hynobiid-like species. We are unable to confirm any of the diagnostic characters listed for *Nuominerpeton aquilonaris*^16^, but the unsculptured skull bones, body proportions (axial length/limbs), vertebral shape, and visible limb morphology (e.g. humerus wider distally than proximally; well ossified carpal and tarsal elements) of the ‘gut salamander’ are consistent with *Nuominerpeton*, and we provisionally refer it to that taxon. The nine specimens of *Nuominerpeton* previously recovered from Pigeon Hill^16^ included four larvae (SPL 33.9–43.8 mm), one post-metamorphic juvenile (SPL 47 mm), and four adults (SPL 77.7–79.8 mm). The adults have extensive limb ossification compared to the juveniles, with a fully ossified carpus and tarsus. The ‘gut salamander’ is somewhat telescoped and twisted, but it has an SPL of around 78 mm, which would correspond closely to adults of *Nuominerpeton*. Adult status is supported by the fully ossified carpus and tarsus.

## Discussion

The Jurassic and Early Cretaceous deposits of north eastern China have yielded an exceptionally rich and diverse assemblages of plants, invertebrates, and vertebrates, many of which show exquisite preservation of hard and/or soft tissues. As a consequence of this fine preservation, these deposits have also yielded a significant number of specimens with gut contents. These include seeds in some birds (*Jeholornis*, *Sapeornis*^17^), insects and conchostracans in salamanders^4,5^, and several examples of vertebrate predation. As reviewed^18^, the predators (and their gut contents) include the mammal *Repenomamus* (juvenile psittacosaur); the birds *Confuciusornis* and *Jianchangornis* (fish); the non-avian dinosaurs *Sinosauropteryx* (mammal), *Sinocalliopteryx* (*Confuciusornis*, *Sinornithosaurus*, indet. ornithischian dinosaur), and *Microraptor* (enantiornithine bird); the choristodere *Hyphalosaurus* (fish); and the lizard *Yabeinosaurus* (fish). Previous authors^17,19–21^ inferred that the Jehol amphibians fed predominantly on insects and worms, and this would be a reasonable inference for *Genibatrachus*, given the many small, closely packed, teeth. However, frogs are opportunist feeders that take a range of foods, as demonstrated by YLSNHM01088.

**Figure 4:**
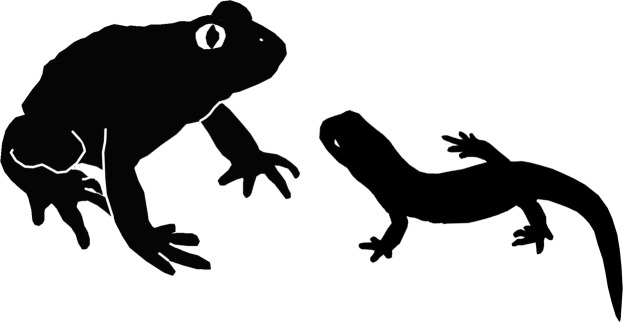
Reconstruction of the frog and salamander, roughly to scale, in silhouette. The frog image is based on the extant *Alytes*, and the salamander image on the extant *Hynobius*.

Extant terrestrial salamanders are eaten by a variety of predators including snakes, birds, small mammals, turtles, frogs, and other salamanders^22,23^, and they can represent a significant prey biomass in some environments^23^. Defence mechanisms include aposematic colouring, posturing, and unpleasant or toxic skin secretions^24^, but whether these were used by early salamanders is conjectural. The salamander skeleton within YLSNHM01088 is largely intact with its bones in association. This suggests it had been caught and swallowed whole, apparently tail first given the position of the skeleton (with the head lying proximally in the gut) and presumably still alive, not long before the frog died and was buried. Predator and prey were of comparable size (Fig. 4), and although the salamander was more gracile in its build, there must have been a struggle.

## Methods

The specimen was collected from the Pigeon Hill locality and is accessioned in the collections of the Yingliang Stone Nature History Museum (YLSNHM), Nan’an, China. The specimen was digitally imaged at high resolution; the images of the part and counterpart blocks (Fig. 2) were then imported into Photoshop to digitally dissect the salamander skeleton from the background (Fig. 3a,b); and the bones from the two blocks were superimposed to form the composite (Fig. 3c).

The map in Fig. 1 was created with Surfer™, Version 7^25^ and ArcGIS™, Version 9.2^26^.
